# Testicular Seminoma After Testicular Torsion Presenting With a Contralateral Inguinal Lymph Node Metastasis: A Case Report and Literature Review

**DOI:** 10.7759/cureus.106906

**Published:** 2026-04-12

**Authors:** Birgitt Allaert, Kurt Geldhof, Karl Lesage, Maarten Albersen, Siska Van Bruwaene

**Affiliations:** 1 Urology, AZ Groeninge, Kortrijk, BEL; 2 Oncology, AZ Groeninge, Kortrijk, BEL; 3 Urology, UZ Leuven, Leuven, BEL

**Keywords:** germ cell tumors, inguinal lymph node metastasis, seminoma, testicular malignancy, testicular torsion, urology emergency

## Abstract

We report the case of an adult male developing a right lymphadenopathy 18 months after being diagnosed with seminoma of the left testis and with a prior history of torsion of the left testis. This is a rare presentation of metastasized germ cell tumor, making it even more exceptional due to the contralateral localization and preceding testicular torsion.

A 44-year-old male was diagnosed with non-metastatic seminoma of the left testis, after having a detorsion and bilateral orchiopexy for torsion of the left testis four years earlier. Eighteen months after an inguinal orchiectomy, he presented with a swollen mass in the right inguinal region. No other lesions were visible on computed tomography (CT) thorax-abdomen and magnetic resonance imaging (MRI) of the brain at the time of presentation. Resection and histopathological examination revealed an inguinal lymph node metastasis of the seminoma. In the meantime, the patient developed four subcutaneous nodules on the abdominal wall, but an additional biopsy was negative. Postoperatively, a curative treatment with three cycles of bleomycin, etoposide, and cisplatin (BEP) was suggested.

Contralateral lymph node metastases from seminoma are extremely rare but may be explained by altered lymphatic drainage following prior scrotal or inguinal surgery. We therefore advise including a bilateral inguinal clinical examination within this specific patient population. Although no association has been established between a history of testicular torsion and the risk of developing germ cell tumors, clinicians should consider potential testicular malignancy in elderly male presenting with a testicular torsion.

## Introduction

Testicular cancer is a relatively rare malignancy, affecting male subjects between 15 and 40 years old predominantly [1]. It accounts for about 1% of all male cancers and is the most common malignancy in young men [2]. Between 90% and 95% of testicular cancers are germ cell tumors (GCTs) [3]. Most patients present with stage I disease confined to the testis (70-75%) or stage II disease with metastases limited to the retroperitoneal lymph nodes (approximately 20%). Five-year survival rates are excellent, reaching 99%, 92%, and 85% for stage I, II, or III, respectively [3]. GCTs are further classified into seminomas and non-seminomatous germ cell tumors (NSGCTs), based on their embryological origins and differentiation pathway [1,3]. The first type is more common in older men, the latter is more common in younger patients and comprises several histological subtypes, including choriocarcinoma, embryonal carcinoma, yolk sac tumor, teratoma, and mixed tumors [2]. Clinically, seminomas tend to exhibit a more indolent course, while NSGCTs have a more aggressive behavior with a higher propensity for lymphatic or hematogenous spread [4].

Of those two, seminoma is the most common GCT affecting the testis (55-60%). At the time of diagnosis, regional or distant metastasis are present in about 15% of pure seminomas [5]. Testicular malignancies typically metastasize in a predictable pattern through the lymphatic system. Tumors arising from the left testicle most commonly spread to the para-aortic lymph nodes, whereas right-sided tumors initially metastasize to the retroperitoneal nodes, with subsequent spread to the inter-aortocaval lymph nodes afterwards [6]. However, prior surgical procedures may disrupt normal lymphatic drainage, resulting in atypical patterns of lymphatic spread [5,6].

We report a case of a 44-year-old man with right inguinal lymph node metastasis after left inguinal orchiectomy for seminoma, with a prior history of surgery for a torsion testis on the left side. This case report aims to perform a comprehensive literature review to examine whether a history of testicular torsion is associated with an increased risk of developing testicular cancer, and to critically evaluate proposed biological or clinical mechanisms that might account for developing contralateral metastasis.

## Case presentation

A 44-year-old Caucasian male presented with an inguinal, painless mass on the right side, 18 months after a left-sided inguinal orchiectomy for a seminoma testicular cancer.

**Figure 1:**
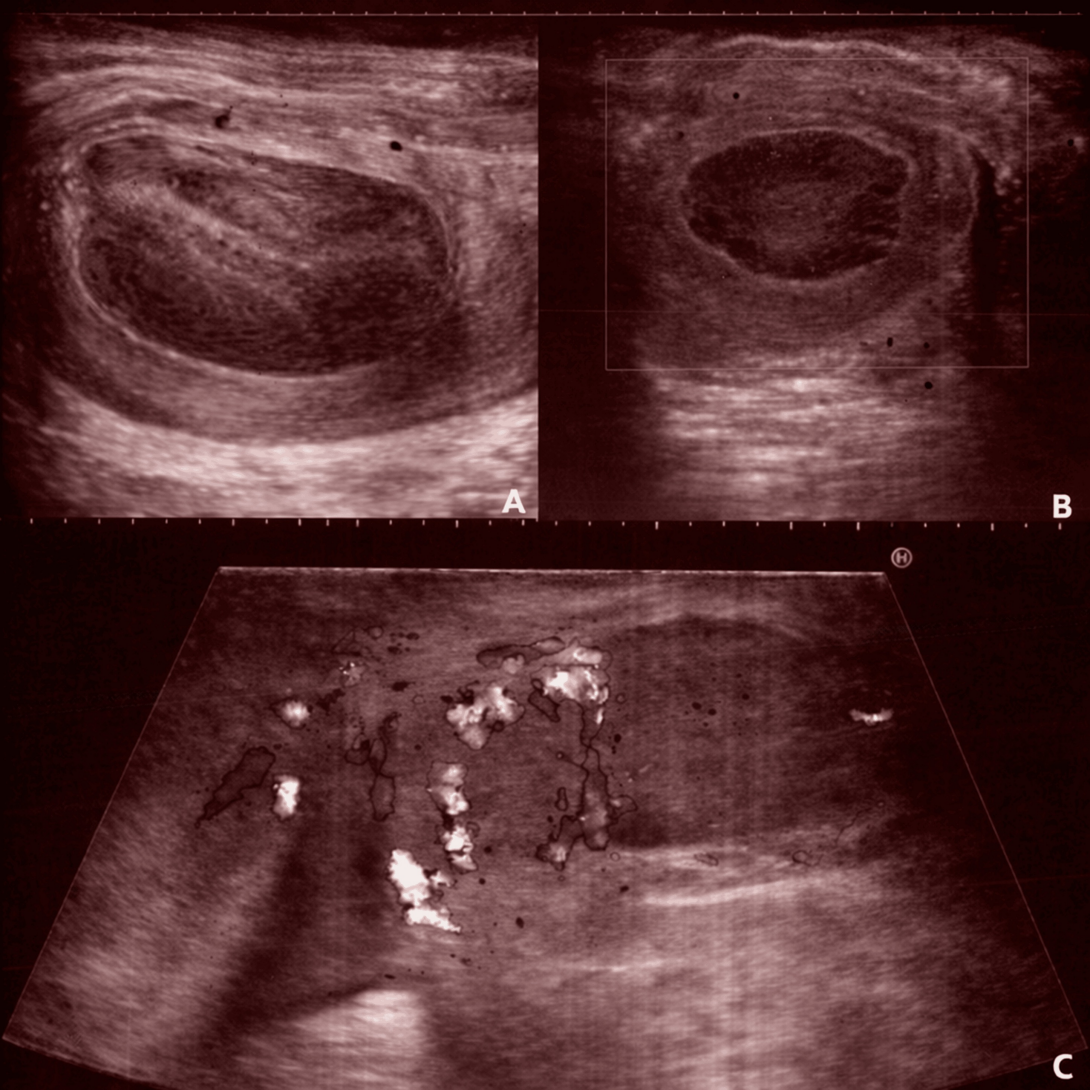
Scrotal ultrasound of the left testicle one month after surgical detorsion and bilateral orchiopexy (A), four months postoperatively (B) and four years later (C).

The patient had a history of subfertility and achieved one live birth via in vitro fertilization (IVF). At the age of 39, he presented with a left-sided testicular torsion. Scrotal ultrasound showed an enlarged left testis with inhomogeneous echogenicity and no vascularization of the testis at the time. Testicular torsion was confirmed during surgical exploration and treated by surgical detorsion of the affected testis, with subsequent visible restoration of vascularization. Concomitant bilateral orchiopexy was performed using Prolene (Ethicon, Inc., Somerville, New Jersey, USA) sutures. The surgical procedure was carried out through a midline incision. Postoperatively, he reported persistent left-sided scrotal pain. Scrotal ultrasound revealed a centrally located, hypo-echogenic area without vascularization, surrounded by peripheral lateral vascular flow (Figure 1A). Conservative management with nonsteroidal anti-inflammatory drugs was initiated, resulting in only minimal recovery of central vascularization. At four months of follow-up, a cystic, necrotic area was visualized on ultrasound, with preservation of peripheral testicular tissue (Figure 1B). The patient was advised to continue regular testicular self-examination.

Four years later, the patient presented with a painful, swollen left testis. Scrotal ultrasound demonstrated hypervascularization of the epididymis and the anterior side of the left testis, with associated posterior hypovascularization and multiple hypoechogenic intratesticular lesions (Figure 1C). An initial diagnosis of acute epididymo-orchitis was made, and treatment with quinolones was initiated. However, due to the absence of clinical improvement, an inguinal orchiectomy was performed. Preoperative tumor markers showed normal levels of human chorionic gonadotrophin (HCG - 1.13 IU/L, reference ranges: <2.0 IU/L) and alpha-fetoprotein (α-FP - 3.42 µg/L, reference ranges: < 7.0 µg/L), but an elevated lactate dehydrogenase (LDH - 318 U/L, reference ranges: 125-250 U/L). Histopathological examination confirmed a pure seminoma staged as pT1, with no lymphovascular invasion and with negative surgical margins. There was no invasion of the rete testis present, but the tumor involved nearly the entire testis with a maximal diameter of 7 cm. Furthermore, there was no evidence of invasion into the scrotal wall or epididymis. Staging computed tomography (CT) scan demonstrated no evidence of lymphadenopathy or metastatic lesions. Due to the tumor size, an adjuvant treatment with a single cycle of carboplatin 7-AUC was given. Clinical, biochemical, and radiological follow-up at 3, 6, 9, and 12 months showed no evidence of recurrence.

**Figure 2:**
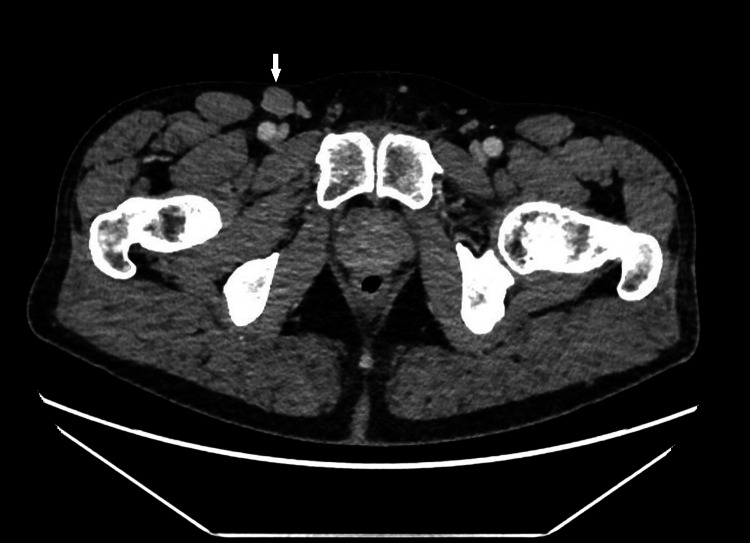
A transversal slice of CT scan of the abdomen revealing a 17 mm right inguinal lymph node (arrow).

Eighteen months after the orchiectomy, the patient presented ambulatory with a painless mass in the right inguinal region. CT thorax and abdomen were performed and showed no other suspicious lesions, aside from the inguinal mass (with a maximal diameter of 17 mm) (Figure 2). All three testicular tumor markers were within normal limits.

**Figure 3:**
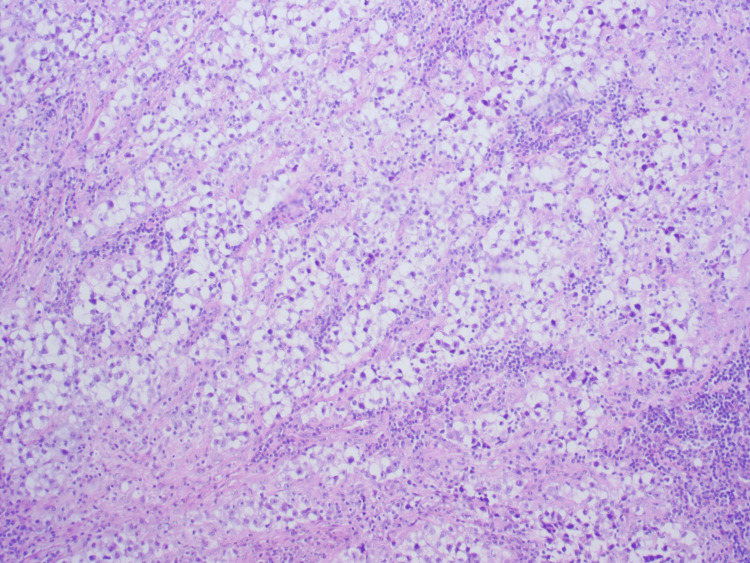
Histopathological examination of the inguinal lymph node demonstrating metastatic seminoma.

Given the progressive growth of the lesion and the previous medical history, a resection of the palpable inguinal mass was performed. No other lymph nodes were resected. Histopathology examination confirmed metastatic seminoma (Figure 3). Subsequent magnetic resonance imaging (MRI) of the brain showed no evidence of additional metastatic disease. 

**Figure 4:**
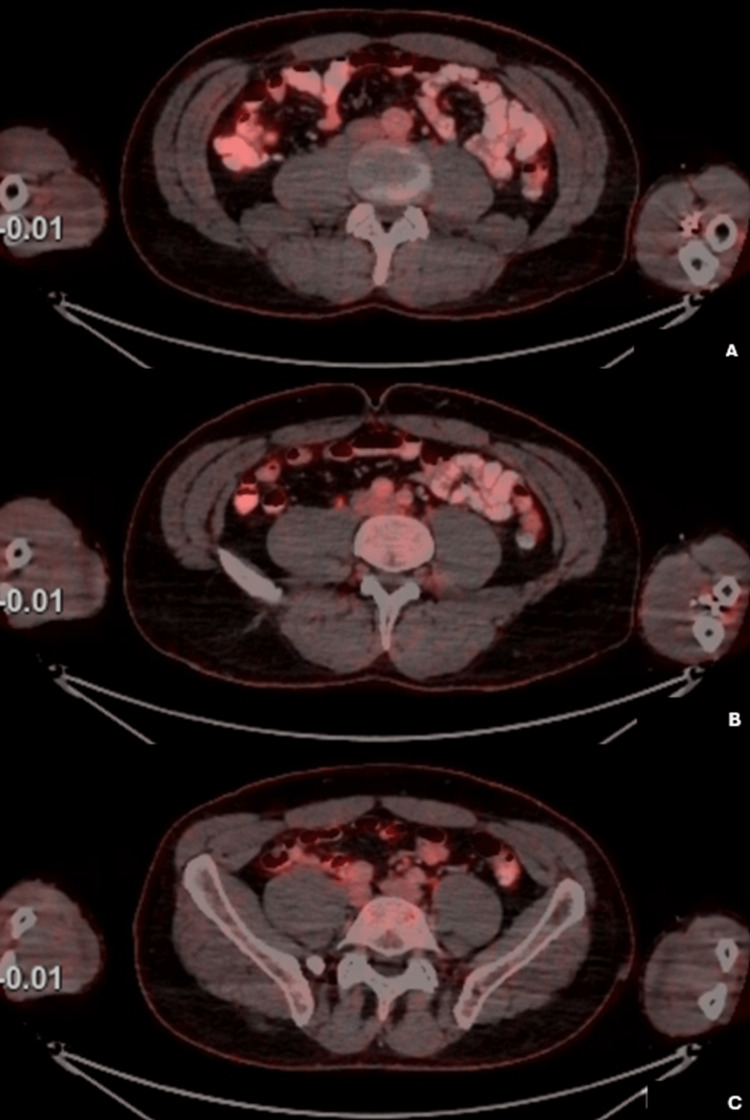
Whole-body 18F-FDG PET-CT revealed no hypermetabolic lesions or abnormal foci of increased FDG uptake on the abdominal wall, and thus no presence of active malignant disease. A: Upper abdominal wall. B: Abdominal wall at the umbilical level. C: Lower abdominal wall. The largest subcutaneous nodule was palpated in the left paraumbilical region. Subsequent surgical excision and histopathological examination confirmed the diagnosis of an angiolipoma. 18F-FDG PET-CT: 18F-fluorodeoxyglucose positron emission tomography-computed tomography.

Of note, between the initial presentation and the oncology consultation, the patient developed four subcutaneous nodules on the abdominal wall, the largest measuring 2 cm paraumbilical. These nodules were not visible on the initial staging CT. To further characterize and exclude possible metastases, a whole body 18F-fluorodeoxyglucose positron emission tomography-computed tomography (18F-FDG PET-CT) was performed, revealing no hypermetabolic lesions suggestive of malignancy (Figure 4).

**Figure 5:**
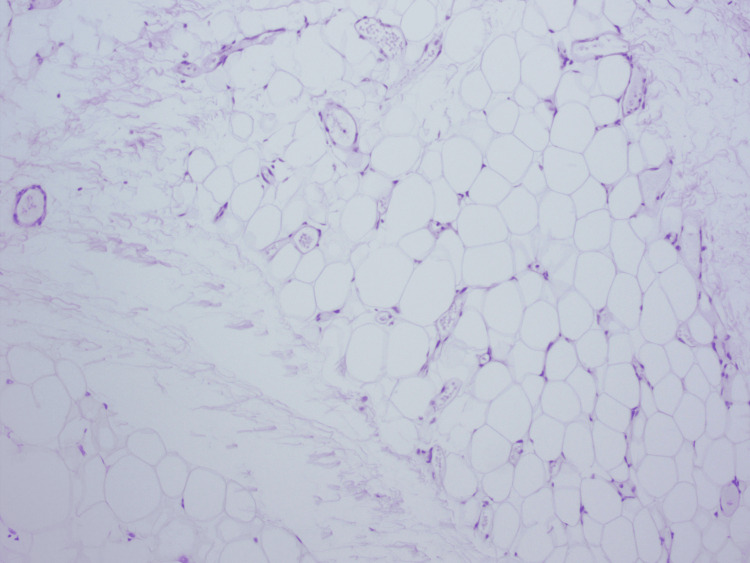
Histopathological examination of one of the subcutaneous nodules confirmed a benign angiolipoma, with no evidence of metastatic seminoma.

For definitive diagnostic confirmation, a biopsy of one of the lesions was obtained. Histopathological examination confirmed a benign angiolipoma, effectively ruling out seminoma metastasis (Figure 5).

According to the International Germ Cell Cancer Collaborative Group (IGCCCG) risk classification [7], the patient was assigned to the good prognosis group. Consequently, the patient was treated postoperatively with a curative regimen of three cycles of bleomycin, etoposide, and cisplatin chemotherapy (BEP), due to the relapse of seminoma following orchiectomy and prior adjuvant chemotherapy.

## Discussion

Primary involvement of the inguinal lymph nodes by seminoma metastases is extremely rare. Secondary metastatic spread to the iliac and inguinal lymph nodes may occasionally occur by retrograde lymphatic dissemination, most often in the setting of bulky retroperitoneal disease [8]. However, in our case, no retroperitoneal metastases were noticeable. Review of the literature shows that the rare presentation of primary inguinal lymph nodes is presumably associated with prior scrotal or inguinal surgery or with direct tumor extension to the epididymis, penetration of the tunica vaginalis with involvement of the scrotal wall, or spread along the vas deferens [8,9]. In this case, no direct tumor invasion to adjacent structures was involved, but the patient had a history of prior scrotal surgery. However, the exact incidence of inguinal lymph node metastasis remains unclear. Daugaard et al. reported an incidence rate of 2% of patients with stage I testicular cancer developing inguinal node metastasis (14 of 695 patients), of whom only two patients were initially diagnosed with seminoma [10]. In this case series, only one patient had a history of prior scrotal surgery, and one showed involvement of the tunica albuginea [10]. Previously, two publications from the 1980s described inguinal metastasis in testicular GCTs. The cohort study by Klein et al. showed 22 patients with inguinal metastases between 1980 and 1984, of whom five were originally diagnosed with a pure seminoma [11]. Between 1968 and 1982, Stein et al. reported 33 patients with testicular tumors of whom four developed inguinal metastases [12]. Yet all 33 patients had NSGCTs; no seminomas were described. However, another case series by Lanteri et al. reported no inguinal lymph node metastasis for patients with testicular carcinoma after prior surgery [13].

Nevertheless, patients with previous scrotal or inguinal surgery tend to be more prone to inguinal lymph node metastasis [9,10,14]. This is thought to result from disruption of normal lymphatic drainage, leading to the formation of collateral pathways between the testicular lymphatics and the afferent lymphatics of the superficial inguinal lymph nodes [9,14]. The latter typically receives afferent lymphatic drainage from the skin of the lower abdominal wall, penis, perineum, buttocks, and scrotum, but after surgical manipulation, this can change drastically [9]. Interestingly, previous reported cases of inguinal metastases from testicular malignancies predominantly describe ipsilateral inguinal lymph node metastasis in almost all cases [8,9]. Contralateral spread is even more rare and, to the best of our knowledge, has only been explicitly reported in four case reports and one retrospective study (Table 1). All case reports involved patients with a history of prior scrotal or inguinal surgery, whereas the single patient reported in the retrospective study had no explicit reported surgical history. In the first case report, Nishimoto et al. refer to experimental studies investigating the injection of dye in dog testis, with expansion of the dye to the para-aortic and ipsilateral iliac nodes in the control dogs, while scrotal surgery resulted in the formation of new lymphatic pathways to contralateral iliac and bilateral superficial inguinal lymph nodes [14]. In the third case report, Rausch et al. suggested that a rare and atypical metastatic route underlying the contralateral metastasis cannot be excluded [15]. This is based on another case report of Lockett et al., describing an alternative metastatic pathway of a testicular tumor via a subepithelial capillary network of the spermatic cord directly into the mesentery, independent of the typical anatomical relationship to vessels of the spermatic cord or vas deferens [16]. In summary, although ipsilateral spread of inguinal metastases is generally expected, clinicians should remain aware that contralateral metastasis may also occur following prior scrotal or inguinal surgery due to altered lymphatic pathways.

**Table 1:** Summary table of reported cases of contralateral inguinal metastasis of testicular carcinoma.

Year	Author	Study type	Inguinal lymph node metastasis	Tumor type	History of scrotal or inguinal surgery
1993	Nishimoto et al. [14]	Case report	Contralateral	Anaplastic seminoma	Yes (bilateral orchiopexy)
2008	Uğur et al. [17]	Case report	Contralateral	Seminoma	Yes (varicocele)
2012	Rausch et al. [15]	Case report	Contralateral	Seminoma	Yes (bilateral open inguinal hernia repair and scrotal vasectomy)
2015	Mao et al. [18]	Retrospective cohort study	1/68 patients with a contralateral inguinal lymph node	Yolk sac tumor (for this particular patient)	Not mentioned for this particular patient
2023	Remil et al. [19]	Case report	First patient: contralateral. Second patient: ipsilateral.	First patient: teratoma. Second patient: seminoma.	Yes (bilateral orchiopexy)

Until today, the detection and management of inguinal lymph node metastasis of testicular cancer following prior scrotal or inguinal surgery remains controversial, due to a lack of robust supportive data [8]. Wheeler et al. support considering routine ipsilateral inguinal lymphadenectomy combined with bilateral retroperitoneal lymph node dissection in NSGCTs who have a history of scrotal or inguinal surgery, even in the absence of retroperitoneal nodal involvement [20]. This recommendation is based on limited evidence derived from small case series, and full-test data remain scarce. In contrast, Mianné et al. concluded that ipsilateral inguinal lymph node dissection is not required in NSGCTs, given the high efficacy of primary or salvage chemotherapy [21]. Yet, they suggest performing additional inguinoscrotal radiotherapy in patients with ipsilateral inguinal metastasis of testicular seminoma [21]. In the case report by Nishimoto et al., the patient underwent an inguinal orchiectomy followed by bilateral inguinal and iliac radiotherapy [14]. At last, Rausch et al. tried to answer the clinical dilemma whether the inguinal solitary metastasis should be classified as an atypical non-regional lymph node metastasis, warranting systemic therapy, or as a regional lymphatic spread amenable to adjuvant radiotherapy [15]. However, despite the absence of typical histological risk factors for occult metastatic seminoma, systemic chemotherapy was recommended; due to the uncertainty regarding the metastatic pathway, undefined radiation dose, and treatment field, along with the risk to the remaining testis by using radiotherapy [15]. To date, no further investigations have been established for either seminomas or the non-seminomas. Overall, there is currently no consensus regarding the optimal management of inguinal lymph node metastasis in testicular cancer following prior scrotal or inguinal surgery. In clinical practice, curative systemic chemotherapy with three or four cycles of bleomycin, etoposide, and cisplatin (BEP) is most frequently given due to the high chemosensitivity of seminomas [15,22]. This is comparable to the treatment of other reported solitary metastasis of testicular seminomas, including duodenal, mesenteric, or prostatic lesions [15,22,23].

On the other hand, our patient initially presented with an acute torsion of the left-sided testis. Postoperatively, he experienced persistent pain, and scrotal ultrasound demonstrated a centrally located hypo-echogenic vascularized area. This finding remained largely stable for over a year, but due to recurrent pain, an inguinal orchiectomy was performed. Nevertheless, testicular torsion is relatively unusual in adulthood [24,25]. A retrospective study of 2016 showed an association between testicular torsion and testicular cancer in 6.4% of the cases [24]. Yet this association remains debatable as no clear pathophysiological mechanisms have been established to explain this relationship [24]. In this present case, it remains uncertain whether the seminoma was already present at the time of the acute torsion, although this is theoretically plausible. Radiologically, seminomas typically appear as non-specific, homogeneous hypo-echogenic lesions on ultrasound, as observed in our case. In this context, this may have been obscured by the presumed necrotic, hypoechoic area resulting from prolonged testicular devascularization [25].

However, if this had been the case, a more extensive tumor burden would be expected, given the four-year interval between initial presentation with testicular torsion and the inguinal orchiectomy. Otherwise, considering the typically indolent nature of seminoma, it is plausible that the tumor burden was still confined to the testis at the time of initial presentation. However, since the tumor measured approximately 7 cm at the time of inguinal orchiectomy, it is important to critically consider whether the tumor may have already been present at the initial presentation with testicular torsion or during subsequent follow-up visits. In particular, the ultrasound performed four years postoperatively should have raised the question of a possible underlying testicular carcinoma.

Assuming that no malignancy was present at the time of the presentation and thus arose secondary to the torsion, the available evidence regarding testicular cancer in patients with a documented history of prior testicular torsion is scarce and largely limited to case reports [4]. In many of these reports, the malignancy was discovered incidentally during surgical exploration for presumed torsion [24]. Notably, there is currently no medical evidence to suggest that a prior episode of testicular torsion independently increases the long-term risk of developing testicular cancer [24]. Nevertheless, urologists should be aware that testicular torsion may occasionally represent the initial manifestation of underlying testicular malignancy, especially in elderly patients [24,25]. In retrospect, consideration of an earlier orchiectomy would have been warranted in this case. However, further research is needed to clarify this association to assess whether testicular torsion may impact testicular malignancy by causing damage to the testicular parenchyma [4].

Awareness of atypical metastatic localization of GCTs remains important. In patients with a history of scrotal or inguinal surgery, we should be aware of the rare possibility of inguinal lymph node metastasis. As this case represents only the sixth reported case of explicitly documented contralateral lymph node metastasis, we want to emphasize the importance of acknowledging the potential for contralateral or bilateral metastatic inguinal involvement after prior surgery. We therefore recommend a thorough bilateral clinical examination of the inguinal region during follow-up of patients with GCT and prior scrotal or inguinal surgery. Furthermore, the possibility of a testicular malignancy should be considered in elderly patients presenting with a testicular torsion. However, to this date, no clear association has been established between testicular malignancy and a prior history of testicular torsion.

## Conclusions

Inguinal lymph node metastases are rare, especially those located on the contralateral side of the affected testis. However, scrotal or inguinal surgery altering the lymphatic drainage of the testis can exhibit the possibility of atypical inguinal node metastasis on both sides. To date, there is no consensus on the follow-up of patients with prior scrotal or inguinal surgery. Yet, we would recommend a bilateral clinical follow-up with subsequent fine needle aspiration or resection of new atypical inguinal lesions when in doubt. Current literature about optimal treatment management remains controversial due to the lack of robust data. Nevertheless, since these lymph nodes are assumed to be metastatic, a curative treatment of chemotherapy with three or four cycles of BEP is considered appropriate due to the high chemosensitivity of seminomas. At last, awareness about the possibility of testicular malignancy in elderly men presenting with testicular torsion remains important. When in doubt, a biopsy or inguinal orchiectomy should be considered.
